# The life cycle of cancer-associated fibroblasts within the tumour stroma and its importance in disease outcome

**DOI:** 10.1038/s41416-019-0705-1

**Published:** 2020-01-29

**Authors:** Elisa D’Arcangelo, Nila C. Wu, Jose L. Cadavid, Alison P. McGuigan

**Affiliations:** 1 Institute of Biomaterials and Biomedical Engineering, University of Toronto, Toronto, ON Canada; 2 Department of Chemical Engineering and Applied Chemistry, University of Toronto, Canada 200 College St., Toronto, ON M5S 3E5 Canada

**Keywords:** Cancer microenvironment, Tumour heterogeneity, Drug development

## Abstract

The tumour microenvironment (TME) determines vital aspects of tumour development, such as tumour growth, metastases and response to therapy. Cancer-associated fibroblasts (CAFs) are abundant and extremely influential in this process and interact with cellular and matrix TME constituents such as endothelial and immune cells and collagens, fibronectin and elastin, respectively. However, CAFs are also the recipients of signals—both chemical and physical—that are generated by the TME, and their phenotype effectively evolves alongside the tumour mass during tumour progression. Amid a rising clinical interest in CAFs as a crucial force for disease progression, this review aims to contextualise the CAF phenotype using the chronological framework of the CAF life cycle within the evolving tumour stroma, ranging from quiescent fibroblasts to highly proliferative and secretory CAFs. The emergence, properties and clinical implications of CAF activation are discussed, as well as research strategies used to characterise CAFs and current clinical efforts to alter CAF function as a therapeutic strategy.

## Background

Conceptualising cancer as an exclusively tumour cell-centric disease is no longer an effective approach for identifying novel therapeutic targets.^1^ Invasive cancer in particular, has been described as a ‘derangement in the proper sorting of cell populations, causing a violation of normal tissue boundaries’,^2^ and requires a two-way, tumour-permissive relationship between host stroma and tumour epithelium. The stromal components of a tumour have thus graduated in our appreciation from bystanders, to enablers, to indispensable and determinant players with respect to disease evolution and response to therapy.^3^ Thanks mainly to more sophisticated in vivo models and analysis techniques,^4,5^ a focused effort is currently underway to consider the tumour-cell phenotype as an emergent property of both the tumour microenvironment (TME) and the activity of stromal cell populations that surround and infiltrate the tumour.

The stromal content of solid tumours can vary significantly, both in amount and composition; in certain tumour types, such as pancreatic, stomach, colon and breast cancer, the stroma contributes 60–90% of the total tumour mass.^6–8^ Tumour stromal tissue comprises fibroblasts, endothelial and immune cells, and extracellular matrix (ECM) components, which all differ markedly from their quiescent counterparts in homoeostatic tissues. Fibroblasts are of particular interest owing to their extensive matrix-synthesising and matrix-remodelling capacities, both of which are pivotal for establishing an invasion-permissive TME.^9^ In fact, levels of fibroblast markers within tumours are known to be predictive of disease outcome, with the number and properties of fibroblasts in the tumour stroma influencing overall survival, time to recurrence and resistance to therapy.^10,11^ Targeting fibroblast function for therapeutic benefit is therefore an area of intensive research.^11^

The term ‘fibroblast’ describes a heterogeneous group of spindle-shaped cells of mesenchymal origin with a spectrum of phenotypes characterised by varying degrees of contractility, ranging from the resting fibroblast to the activated myofibroblast observed in wound healing.^12^ Fibroblasts that have become activated during wound healing are referred to as ‘myofibroblasts’, while those activated in the context of tumours are called carcinoma-associated fibroblasts (CAFs) or peritumour fibroblasts.^13^ Though different in nomenclature, activated fibroblasts display analogous marker expression and very similar phenotypes, with the important distinction that in contrast to acute inflammation, fibroblasts in chronic inflammation and tumour tissue fail to inactivate.^10^ A great number of studies have identified the signalling mechanisms by which CAFs influence tumour cell properties (we refer the reader to several comprehensive reviews),^11,14^ and it has become clear that CAFs play a pivotal role in the TME. However, our understanding of the diverse properties of CAFs throughout the different stages of tumour progression remains incomplete. The function of CAFs has remained especially elusive in light of their heterogeneity and different effects on tumour development; while their pro-tumorigenic role has been well documented across tumour types,^15^ CAFs have also been reported to restraining tumour growth in several studies.^16–18^

In this review, we describe the dynamics of fibroblast activation in acute and chronic wounds, focusing on solid cancers. Most studies investigating the CAF–tumour cell relationship concentrate on carcinomas at the invasive stage, but we attempt to look before and after this stage to provide insights into the wider life cycle of fibroblasts located in the tumour stroma. Specifically, we describe the properties of fibroblasts as they progress from their resting state in tissue homoeostasis to the different magnitudes of activation occurring during early, invasive and metastatic tumour growth. Finally, we consider the clinical relevance of the activated fibroblast phenotype and opportunities for targeting fibroblast activation therapeutically.

## Fibroblasts in homoeostasis

During tissue homoeostasis, epithelial cells are strictly segregated from fibroblasts in the adjacent connective tissue^19^ by the basement membrane (BM), a specialised ECM structure that presents the biophysical cues necessary for cell attachment, polarity and cell movement within and across tissue compartments.^20,21^ Resting or quiescent fibroblasts are dispersed as single cells within the stromal tissue compartment and are found proximal to the BM, where they orient themselves in the direction of matrix fibres, but have no direct association with the BM.^22^ During homoeostasis, quiescent fibroblasts show minimal metabolic and transcriptional activity but play a pivotal role in defining the differentiation status of adjacent epithelia via the secretion of specific signalling factors, including among others Wnt and bone morphogenic proteins,^23,24^ and the production and organisation of matrix proteins, including collagens, fibronectin and elastin, at a minimal rate, to maintain a compliant, yet tension-resistant BM.^4^

Although the structural characteristics of quiescent stroma are relatively well defined, the molecular characteristics of quiescent fibroblasts have remained elusive. From a collection of several markers that are not exclusive nor obligate to fibroblasts across tissues, fibroblast-specific protein 1 (FSP-1), a cytoplasmic calcium-binding protein, has been proposed as the most accurate marker for resting fibroblasts to date (even though FSP-1 is also found on cancer cells that have undergone EMT,^25^ as well as being expressed on macrophages^26^); other markers include vimentin, filamin A and Thy-1/CD90.^4,27^ Consequently, the quiescent fibroblast is generally considered to be a fibroblast that does not express the markers observed in activated fibroblasts, as defined primarily from studies of fibrosis and wound repair. As such, perhaps the most apt characterisation of a quiescent fibroblast is that of a mesenchymal cell with the potential to become activated by growth factors, and drastically increase its contractility, proliferation and secretory phenotype in response to an appropriate stimulus.^4^ Note that the lack of data specific to resting fibroblasts might be attributable to two factors. First, the focus is typically placed on their tissue-specific, activated counterparts (myofibroblasts or CAFs).^4^ Second, the inability to establish fibroblast cultures with a truly ‘resting’ phenotype has hampered the investigation of these cells in vitro; although fibroblasts can readily be cultured in vitro,^4^ they become at least partially activated owing to the properties of culture plastic, which is reminiscent of stiffened, fibrotic ECM.^28,29^

## Fibroblasts in non-homoeostatic conditions

### Wound healing

**Fig. 1:**
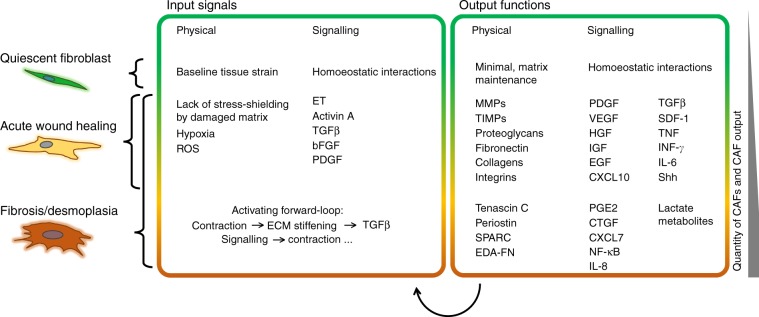
Overview of the signal inputs and functional outputs of fibroblasts activated across the fibroblast activation spectrum. Fibroblasts respond to physical cues and chemical signalling factors in the tumour microenvironment (TME) (‘input signals’) by producing a number of molecules that signal in an autocrine and paracrine fashion, as well as by altering the physical properties of the TME (‘output functions’). In contrast to acute wounds, fibroblasts in desmoplasia establish a self-activating and perpetual feedback loop (bottom arrow), which forms the basis of their pro-tumorigenic capacities. Moving from the quiescent phenotype (green, top), to the CAFs in desmoplasia (orange, bottom), the number of known inputs and outputs increases cumulatively. *ROS* reactive oxygen species, *ET* endothelin, *TGF-β* transforming growth factor β, *bFGF* basic fibroblast growth factor, *PDGF* platelet-derived growth factor, *MMPs* matrix metalloproteinases, *TIMPs* tissue inhibitors of metalloproteinases, *VEGF* vascular endothelial growth factor, *HGF* hepatocyte growth factor, *IGF* insulin-like growth factor, *EGF* epidermal growth factor, *CXCL* CXC motif chemokine ligand, *SDF-1* stromal-cell-derived factor-1, *TNF* tumour necrosis factor, *IFN-γ* interferon-γ, *IL* interleukin, *Shh* sonic hedgehog, *SPARC* secreted protein acidic and rich in cysteine, *EDA-FN* EDA-containing cellular fibronectin, *PGE2* prostaglandin E2, *CTGF* connective tissue growth factor; *NF-κB* nuclear factor κB.

The acute inflammation that occurs during wound healing is a classic example of the loss of the homoeostatic ‘separation’/sorting behaviour of epithelial and stromal-cell populations and is characterised by the appearance of activated fibroblasts (myofibroblasts); indeed, much of what we know about fibroblast activation was first observed in the context of acute inflammation. During wound healing, the goal of fibroblast activation is to re-establish the pre-injury tissue architectural and homoeostatic states,^4,12^ and accordingly the fibroblast content in wounds increases via proliferation and recruitment.^30^ Fibroblasts become activated (as myofibroblasts) to perform a number of functions. They regulate the de novo deposition of matrix proteins and remodelling of the existing matrix via the secretion of matrix-degrading enzymes (e.g. matrix metalloproteinases) and through enhanced contractility.^31^ Their contractile apparatus also enables them to regulate interstitial fluid pressure.^24^ Through a plethora of secreted factors, fibroblasts can also interact with other cell types,^32^ in order to re-vascularise the healing tissue, and furthermore they secrete and absorb metabolites to rebalance the tissue niche.^12^ A key functional difference between fibroblasts and myofibroblasts therefore involves the cell secretome, with myofibroblasts demonstrating an increased production of proteins, including signalling molecules. Furthermore, on a molecular basis, while resting fibroblasts are characterised by the absence of activation markers and markers typical for other cell types,^12^ myofibroblasts acquire, in addition to FSP-1, and among others, the expression of α-smooth muscle actin (α-SMA), desmin, fibroblast activation protein (FAP), discoidin domain-containing receptor 2 (DDR2), collagens (e.g. collagen 1a1) and fibronectin, as well as numerous other growth factors and receptors, cytokines and ECM components,^10^ many of which are listed in Fig. 1.

### Tumours

CAFs in tumours acquire the same features as do myofibroblasts in inflammation, and no systematic stratification or differences have been identified between these fibroblast populations. Figure 1 summarises the similarities and differences in functional properties of fibroblasts as they transition from quiescence to acute inflammation (myofibroblasts) and chronic inflammation/tumours (CAFs), and Table 1 describes the common functional assays used to assess the properties of activated fibroblasts; for a more exhaustive list of common assays to assess invasion, refer to ref. ^33^ A crucial difference between chronic wounds/tumours and acute inflammation however, is the failure to abate the fibrotic response in the former. Whereas the properties of myofibroblasts lead to the re-establishment of tissue architecture in acute inflammation, the acquisition of these same properties over prolonged periods in chronic wounds/tumours leads to further disorganisation of tissue architecture and tissue desmoplasia, a term that specifically denotes an increase in stromal cells and matrix components within an injured tissue.^3^

**Table 1:** Functional assays to study fibroblast phenotype.

Assay description	Example studies
Transwell invasion assay	
The number of cells that invade through a thin layer of ECM and cross a pore is measured.	CAFs increase the invasiveness of cancer cells by inducing EMT through secreted TGF-β1.^111^
Vertical gel invasion assay	
The invasiveness of cancer cells seeded on an ECM plug is assessed via optical or mechanical sectioning of the gel. CAFs can be easily incorporated into the system.	CAFs are the leading cells in the collective invasion of squamous cell carcinoma cells.^55^
Spheroid/organoid gel invasion assay	
Multicellular spheroids are embedded into a 3D ECM; cell invasion is monitored similarly to the vertical gel assay above.	Collective cancer cell invasion is enabled by heterotypic E-cadherin/N-cadherin adhesions with CAFs.^112^
	Co-culturing pancreatic cancer organoids and pancreatic stellate cells in a 3D ECM results in two CAF phenotypes (myofibroblastic, inflammatory).^113^
Paper-supported culture	
Cellulose scaffolds are seeded with cells suspended in ECM, stacked, cultured to enable invasion and then de-stacked for analysis (e.g. cell counts in different layers).	CAFs enhance the migration of human lung cancer cells.^114^
Microfluidic assay	
These devices allow for the creation of defined tissue compartments and molecular gradients. Cell invasion can be monitored in real time.	CAFs induce the progression of mammary carcinoma in situ to an invasive phenotype.^115^
Fibrin bead assay	
Endothelial cells are cultured on the surface of micro-carrier beads that are embedded in a fibrin gel. Fibroblasts are usually seeded on top of the gel to produce the necessary growth factors for vessel sprouting.	Fibroblast-secreted factors are necessary for angiogenesis and lumen formation.^116^
Gel contraction assay	
An ECM plug is seeded with fibroblasts and is allowed to contract over time. Cell contractility correlates with the change in size of the gel.	Activation of YAP in CAFs is required to promote matrix stiffening.^117^
PDMS wrinkle assay	
Adherent cells are cultured on thin sheets of cross-linked PDMS. Contractile cells exert traction forces that result in visible substrate wrinkling, depending on the stiffness of the PDMS.	Increased α-SMA expression is sufficient to enhance fibroblast contractile activity.^118^

Summary of the most common functional assays to study fibroblast phenotypes and their effect on cancer cells. Of note, much attention in the literature has been given to the pro-invasive role of CAFs on cancer cells, so here we only present the most common invasion assays (for a more exhaustive list refer to ref. ^33^)

*α-SMA* α-smooth muscle actin, *CAFs* cancer-associated fibroblasts, *ECM* extracellular matrix, *EMT* epithelial–mesenchymal transition, *PDMS* polydimethylsiloxane, *TGF-β* transforming growth factor-β, *YAP* Yes-associated protein

During both inflammation and tumour growth, the number of activated fibroblasts at the injured tissue site increases via recruitment of progenitor cells from different sources and proliferation of these cells, as well as tissue-resident fibroblasts,^34,35^ which represent the most immediate pool of cells to be activated into highly proliferative CAFs and recruited to the site of tumour growth. It is unclear whether these different CAF sources give rise to different CAF subpopulations that maintain distinct properties within the tumour over time.

Tissue desmoplasia is common in cases of chronic inflammation and tumours, highlighting the parallels between the two states, and positions the activated fibroblasts as an important mechanistic link between cancer and fibrosis.^36^ Indeed, conditions of chronic inflammation, such as inflammatory bowel disease, hepatic fibrosis or prostatitis, can be pre-emptive of tumour development at these anatomical sites.^37^ Furthermore, because of the similarities between chronic wounds and tumours in establishing fibrotic tissue niches, certain signalling pathways have been identified in both conditions as potential therapeutic targets for decreasing fibrosis and CAF-driven tumour aggressiveness, including the platelet-derived growth factor (PDGF) and transforming growth factor-β (TGF-β) pathways.^38,39^

## The life cycle of CAFs during disease progression

**Fig. 2:**
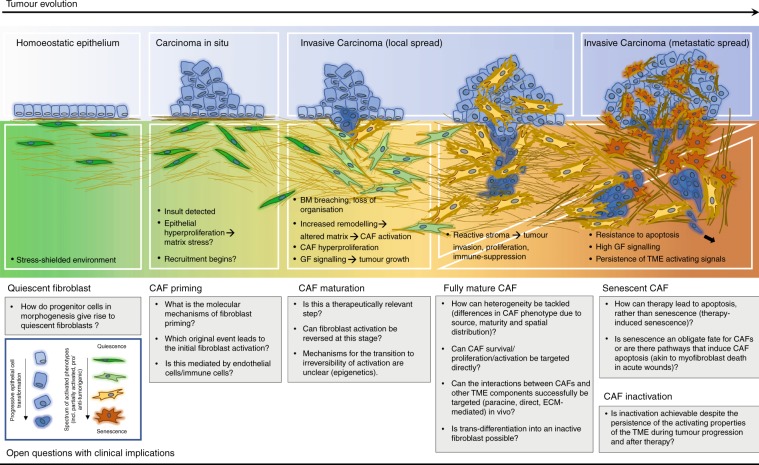
Co-evolution of the tumour and cancer-associated fibroblasts (CAFs) within the tumour stroma. Schematic overview of the changes in tumour cell and CAF phenotypes in the context of an evolving tumour microenvironment (TME). The bottom panel lists open questions in CAF biology with potential implications for therapeutic interventions. The black arrow in the ‘metastatic spread’ panel indicates metastatic spread of tumour cells.

The above discussion has compared the two best-known states of fibroblasts—quiescence versus activation in chronic wounds/tumours. In this section, we focus on the transition that occurs between these states during tumour growth, which is concurrent with the breakdown of local tissue structures and tumour cell invasion. In addition to highlighting the properties of CAFs in invasive disease, we emphasise studies that have provided insight into what precedes and what follows the establishment of a mature CAF and this timeline is illustrated in Fig. 2.

### Fibroblast recruitment during carcinoma in situ

To date, it is unclear whether changes in stromal properties precede or are a result of the hyperproliferation of transformed epithelial cells,^2,40^ and the mechanisms and timing of fibroblast recruitment and activation at the preinvasive stage are poorly understood. The local dysregulation of epithelial signalling and proliferation in an otherwise homoeostatic tissue potentially result in altered chemical signalling to the surrounding tissue stroma and mechanical changes to the BM. Other than paracrine signalling from epithelial cells,^41^ potential sources of fibroblast-activating signals include cues from immune cells that are recruited to the site of epithelial hyperproliferation,^42^ microRNAs (miRNAs) produced by the growing mass of transformed epithelial cells^43^ and mechanical cues arising from a local increase in tissue tension.^44^ These activating signals potentially permeate through the ECM and BM structures of certain porosities (BM pore diameter ranges from 5 nm to 8 µm^21^); alternatively, tumour-derived exosomes carrying, for example, miRNAs or TGF-β family members, have been implicated in establishing tumour-promoting signalling in tumour niches and fibroblast activation.^45,46^ Immune cells, especially macrophages, B cells and regulatory T cells, can directly activate fibroblasts, and increased densities of these cells are found in tumour stroma,^47^ where they accumulate in response to the release of inflammatory mediators by hyperproliferative, transformed epithelial cells.^48,49^ Fibroblast activation might also result from an external insult responsible for initiation of the neoplastic growth itself: one study showed that dermal fibroblasts could be primed by UV radiation, which resulted in an epigenetic loss of Notch signalling, increasing their synthesis of growth factors and capacity to remodel the matrix.^50^

A few specific cancers, such as breast ductal carcinoma in situ (DCIS), offer the opportunity to explore fibroblast status prior to BM breaching. For example, one study examined the gene expression signatures of stromal cells in DCIS versus invasive cancer^51^ and identified a 66-gene signature common to fibroblasts from invasive breast cancer and a subset (40%) of DCIS tissues, suggesting that fibroblast activation occurs at the preinvasive tumour stage. Analogously, a comparison of the expression of angiogenic genes between the stroma of non-malignant, preinvasive, invasive and metastatic breast tissues revealed a clustering of these genes in all but the healthy tissues.^52^ Furthermore, a study specifically investigating the appearance of α-sma+ myofibroblasts in normal, benign and malignant breast tissue observed a shift from the presence of exclusively CD34+ fibrocytes in normal tissue to the emergence of α-sma+-reactive myofibroblasts in benign lesions and invasive tumours concurrent with the loss of the CD34+ cell populations.^53^ These results further suggest that stromal-cell activation can occur at the preinvasive stages of the disease, and that the tumour stroma as a whole is then ‘primed’ for the subsequent stages of tumour development.

### CAF maturation during basement membrane breaching and local invasion

The transition from in situ to locally invasive disease is a crucial step for tumour growth and requires breaching of the BM. This process is carried out actively by the tumour epithelium, immune cells and CAFs^54^ and exposes these cell populations to each other, allowing for direct contact and increased short-range signalling between them. CAFs are capable of digesting BM components and might therefore be responsible for mediating significant breaching of this structure.^2^ Previous studies, for example, have revealed the capacity of CAFs to break down BM via matrix-metalloproteinase (MMP)-mediated digestion to generate ‘tracks’ that were subsequently occupied by invading carcinoma cells.^55^ Moreover, CAFs that were presented with murine mesentery (a BM substitute) induced cell permeability in the membrane via an MMP-independent mechanism; they remodelled the matrix by applying contractile forces, creating gaps between BM fibres that allowed cancer cells to pass through.^56^ Importantly, a caveat of such studies is the use of fully mature CAFs sourced from tumours that were already invasive and our appreciation of the contribution of stage-matched CAFs to BM breaching during the transition to locally invasive tumour growth is limited.

As a consequence of the changes in tissue structure (ECM composition and organisation), tissue architecture/composition (intermingling of CAFs with other cellular fractions) and activated fibroblast secretome during local tissue invasion (see Fig. 1), the parameters that dictate the gradients of CAF-produced signals are likely to change to more readily influence other TME cellular components, leading to an increasingly activated TME. It is useful at this point to draw a parallel with fibroblast activation during acute wound healing, a process in which the temporal dynamics of activation have been well defined, and a two-stage model of myofibroblast differentiation has been proposed.^57^ In inflamed tissue, fibroblasts increase their production of stress fibres (composed of cytoplasmic actin) and organised fibronectin on their cell surface, due to the mechanical tension present. These features promote the transition of a normal fibroblasts to an intermediate stage known as a protomyofibroblast. This cell type is characterised by an enhanced contractile capacity compared with quiescent fibroblasts, and has been observed in 2–4 day-old wounds within the granulation tissue (the new connective tissue created to seal the wound).^58^ Protomyofibroblasts have not yet incorporated α-SMA into their stress fibre network in order to generate the potent traction forces on the surrounding matrix that are characteristic of a mature myofibroblast.^31^ However, continued mechanical tension induces the production of specific splice variants of fibronectin, mainly ED-A and ED-B, which have been shown to guide transdifferentiation of protomyofibroblasts into myofibroblasts through poorly understood mechanisms.^58^ Concurrently, autocrine or paracrine signalling through the TGF-β pathway results in the synthesis of α-SMA and collagen type 1, ultimately giving rise to fully mature myofibroblasts.^57^

In contrast to the process described above, the temporal dynamics of fibroblast activation during tumour progression have yet to be firmly pinpointed. However, it is reasonable to surmise that a fibroblast entity equivalent to the protomyofibroblasts found in acute wounds might exist, which can be thought of as an intermediately activated fibroblast with lower contractility that arises at some point during the early stages of tumorigenesis. The further activation of such intermediates could then be achieved by the several CAF-mediated signalling pathways known to perpetuate further CAF activation.^10^ For example, the production of TGF-β and stromal-cell-derived factor-1 (SDF-1, also known as CXC motif chemokine 12 [CXCL12]), by CAFs results in further fibroblast activation.^59^ Similarly, CAF-mediated expression of leukaemia inhibitory factor (LIF) is responsible for initiating, within CAFs themselves, the constitutive activation of Janus kinase 1 (JAK1)/signal transducer and activator of transcription 3 (STAT3) signalling via p300-mediated histone acetylation, resulting in enhanced CAF contractility and consequent ECM remodelling. These properties lead to further CAF activation, consequent tumour-cell invasion and further breakdown of tissue architecture.^60,61^

As exemplified above, epigenetic regulation is considered, for a number of reasons, to be a major contributor to CAF activation within the TME.^62^ First, fibroblast transition from the quiescent to the activated state is not based on the acquisition of genetic errors (CAFs are mostly considered genetically stable, although debate on this topic continues^35,63^). Second, the epigenetic status is heritable and microenvironmentally induced, and is therefore likely to fit the dynamics of fibroblast activation.^64^ For example, TGF-β and other growth factor signals result in global changes in the methylation status of fibroblast genes.^62,65^ In lung cancer stroma, *SMAD3* was one of the genes found to be silenced by hypermethylation, which elicited a hyper-responsiveness of CAFs to TGF-β signalling, leading to an increased expression of wound-response genes such as *COL1A1*, thereby linking TME methylation to increased ECM deposition by CAFs.^66^

During local invasion, the physical interaction and bidirectional movement between epithelial and stromal tissue components that follows BM breakdown creates a ‘zone of mixing’, from which metastatic cells eventually emerge.^2^ These mixing zones attract a continuous supply of fibroblast progenitors from different cell pools (see above), which differentiate/become activated, proliferate and infiltrate the tumour mass.^10,67^ Such interface zones of tumour cells and tumour stromal-cell populations are arguably sites in which intense TME remodelling occurs, and which are characterised by unique functionalities and marker expression, including markers of invasion or EMT in epithelial cells.^68,69^ Utilising a clonal tracing strategy, a recent study found that tumour cells with cancer stem cell properties were located along the tumour margin and regulated by CAF-secreted osteopontin.^70^ With respect to CAFs, a study investigating the differences between fibroblasts at increasing distances from the surgical tumour margin identified increased pro-proliferative and EMT-inducing capacities between all CAFs compared with fibroblasts taken at least 10 mm from the tumour margin.^71^ More importantly, however, CAFs sourced from the interface zone were more potent inducers of tumour progression than CAFs present within the tumour core, as assessed in vitro, indicating that interface CAFs possessed a pronounced tumour modulatory capacity.^71^

### Mature CAFs during local and metastatic spread

The complete CAF response to the presence of tumour epithelia and remodelled matrix takes place in tumour–stroma mixing zones. The local tissue architecture becomes highly distorted by CAF infiltration, with aberrant de novo fibrinogenesis and matrix cross-linking (via CAF-secreted lysyl oxidase).^72,73^ The consequent rise in tissue tension continues to catalyse the activation of CAFs, which enter the feed-forward loop of contractility, ECM stiffening and TGF-β production.^31^ Analyses of CAFs present in invasive carcinomas have revealed the presence of diverse activation markers and associated functions, which might reflect differences in CAFs across tissues, differences in the proportions and origins of CAF progenitor cells recruited, and a possible spectrum of CAF ‘maturity’ within the TME.^74^

A number of classifications of CAFs have been recently proposed. At a minimum, and based on the most polarising effects of the CAF phenotype, a stratification between tumour-promoting and tumour-restraining CAFs has been identified.^18,75^ The tumour-promoting activities classically identified in CAFs across tumour tissues include promoting tumour cell survival, growth and stemness, as well as metastatic capacity.^10^ Furthermore, CAFs have been implicated in affecting chemoresistance via specific CAF paracrine signalling (e.g. interleukin [IL]-6 ^76^ or plasminogen activator inhibitor [PAI-1] ^77^), as well as CAF-mediated drug scavenging and the establishment of a biophysical ECM barrier (the latter two were more commonly identified in highly desmoplastic solid tumours such as PDAC). These CAF activities hamper drug delivery and reduce drug efficacy.^78,79^ CAFs with tumour-restraining activity, on the contrary, have been named the ‘F1’ subtype, and a landmark study has shown their tumour-restraining qualities in the pancreatic tumours in a transgenic mouse model, where depletion of α-SMA+ CAFs resulted in tumours with enhanced aggressiveness via an upregulation of CD4+ regulatory T cells.^16^ Meflin, a marker of perivascular mesenchymal stromal cells, was recently proposed as a marker of tumour-restraining CAFs in pancreatic cancer.^18^ By contrast, the ‘F2’ CAF phenotype denotes pro-tumorigenic fibroblasts, which have been the focus of most studies and described above.^75^ It remains unclear whether a choice exists in different organs between an F1 and F2 fate, or how the F1 phenotype persists in certain cases, and whether a switch from an F2 phenotype to an F1 phenotype is possible. Further stratification between CAF subtypes has been provided, again in pancreatic cancer, by Tuveson et al., who revealed the presence of CAF populations termed ‘iCAFs’, ‘myCAFs’ and antigen-presenting CAFs, which have distinct roles in direct interactions with tumour cells, paracrine signalling and modulation of the immune niche, respectively.^80,81^ Other studies have further assessed CAF heterogeneity at the single-cell level.^82,83^ Based on the expression patterns of specific sets of genes in breast cancer, distinct but overlapping clusters of ‘protomyofibroblasts’, ‘ECM-regulating myofibroblasts’ and ‘secretory myofibroblasts’ were identified and a fibroblast differentiation model could be constructed, which suggested a hierarchical organisation of fibroblast populations.^82^ RNA-Seq revealed further CAF heterogeneity with CAF subgroups expressing independent prognostic biomarkers and arising through EMT from distinct cellular sources, including tumour cells, resident fibroblasts and perivascular cells.^83^ Overall, this heterogeneous impact of CAFs on tumour progression might depend on the properties of the tumour cells themselves, or be reflective of tumour stage.^35^

### Fibroblast senescence

Another stage in the life cycle of CAFs that has been identified is senescence—that is, the decline of the proliferative and differentiation capacity of CAFs and CAF progenitors, with a concurrent increase in secretory capacity.^84,85^ In terms of the positioning of senescence along the CAF life cycle timeline, no information is available; however, in vitro, senescent CAFs are encountered after several culture passages—particularly rapidly when the cells were obtained from older tissues.^4^ Senescence is a process associated with cellular ageing that results from telomere dysfunction and senescence stimuli, such as the accumulation of reactive oxygen species and DNA damage,^86^ eventually leading to growth arrest in G1, an enlarged morphology in vitro, non-responsiveness to mitogens and resistance to apoptosis. Importantly, apoptosis and senescence are viewed as alternative cell fates, and the balance between them appears to be cell specific.^87^ In CAFs, senescence has a reported dual effect: it arrests the proliferation of CAFs in the TME, which could therefore prove beneficial; however, senescent CAFs secrete inflammatory mediators and growth factors, such as IL-8^88^ and MMP-2,^89^ which have pro-tumorigenic effects.^90^ SDF-1/CXCL12, a chemokine present in the secretome of senescent CAFs, was found to mediate tumour cell survival and proliferation both directly and through the recruitment of other TME components.^91^

Because senescent fibroblasts accumulate in ageing tissues, it stands to reason that they could be responsible for conditioning stromal tissue over time, transforming it into a premetastatic niche.^86^ However, the question of whether senescence is the obligate fate of a CAF in desmoplasia remains open. Notably, senescence was observed in fibroblasts cultured in the presence of doxorubicin and paclitaxel, and therapy-induced senescence is a well-known phenomenon,^90^ which highlights an important issue: although it is desirable to therapeutically induce senescence in cancer cells, the senescence of stromal-cell components might induce further inflammation, and ultimately, tumour relapse.^90^

### CAF inactivation and depletion through apoptosis

During wound resolution, the reconstituted ECM eventually takes over the mechanical load of the healing tissue, allowing a large portion of the now stress-released myofibroblast population to undergo apoptosis or inactivation.^92^ Spontaneous myofibroblast inactivation was specifically described in a model of regressing liver fibrosis, which demonstrated that half of the myofibroblast population is inactivated while the remainder undergoes apoptosis.^93^ However, very little is known about the role of inactivation or apoptosis in regulating CAF abundance and function within the TME. CAF loss of function, or at least partial inactivation, has been observed in hypoxic tumours, in which CAFs lose their capacity for contraction and matrix deposition owing to the inhibition of propyl hydroxylase domain protein 2 (PHD2) and stabilisation of hypoxia-inducible factor (HIF)-1α,^94^ which leads to a reduced metastatic potential. The apoptotic sensitivity of CAFs in vivo has also been described and found to be analogous to myofibroblast apoptosis in wound resolution or activation-induced T-cell death, although the underlying basis and timing of the process is unclear.^95^ It remains to be investigated under which circumstances apoptotic sensitivity, rather than senescence, is engaged, and questions surrounding CAF apoptosis, its role in regulating CAF abundance in the TME and its implications in tumour progression are yet to be answered. The addition of all-trans retinoic acid (ATRA)^96^ or the vitamin D analogue calcipotriol^97^ has been shown to inactivate CAFs in genetically engineered mouse models of pancreatic cancer. Beyond these studies however, CAF inactivation and significant levels of apoptosis are not reported in tumours in vivo, most likely as a result of the perpetual activating signals delivered to CAFs by the TME and its dysfunctional matrix,^92^ and possibly also due the induction of a senescent CAF phenotype after a certain, unknown, point in the CAF life cycle. These observations raise the question of whether treatments that aim to achieve CAF inactivation will succeed while the TME ‘wound’ persists.

## Targeting activated CAFs as a therapeutic strategy

While therapies targeting cancer cells directly encounter issues with drug resistance, due to the cells’ genetic instability, the concept of targeting CAFs therapeutically has received growing attention, since CAFs are characterised by relative genetic stability.^35,98,99^ In this section, we summarise the attempts made at targeting or modulating the activated CAF phenotype within the context of the CAF life cycle. Included are compounds in preclinical and clinical stages, which can be roughly categorised, based on the point of activity within the life cycle that they target, as ‘blocking CAF activation’, ‘blocking the CAF hyperproliferative phenotype’, ‘blocking mature CAF function’, ‘inactivating the CAF phenotype’, and ‘directly depleting the CAF population’. Compounds that target CAF-mediated chemoresistance are classified under sections relevant to the mature CAF life cycle and its function. Of note, all five methods ultimately result in a reduced number of CAFs with pro-tumorigenic activity within the TME.

Blocking initial and further CAF activation has been attempted by intercepting signalling pathways between CAFs and/or between cancer cells and CAFs,^100,101^ such as the Rho/Rho kinase (ROCK) pathway and STAT3-mediated activation of insulin-like growth factor type 1 receptor (IGF-1R). As the hyperproliferative phenotype is also observed in fibrosis, anti-fibrotic treatments, such as pirfenidone and tranilast, which target CAF hyperproliferation in maturing and mature CAF populations, have actively made their way towards applications in anticancer therapies.^102–104^ Blocking mature CAF function is currently the focus of a large research area for anti-cancer, CAF-centric therapies that target the cells’ secretome and inhibit growth factor signalling, such as IL-6 and PDGFR signalling.^105,106^ Inactivation of the CAF phenotype attempts to transdifferentiate CAFs towards fates reminiscent of their quiescent counterparts, by leveraging mechanisms involved in apoptosis and/or inactivation, which have been observed to spontaneously occur in fibrotic models, as well as in acute wound healing, and include, for example, calcipotriol (a vitamin D receptor ligand) and minnelide (which de-regulates the TGF-β pathway).^97,107^ Finally, directly depleting the intratumoural CAF population has been accomplished by targeting cell surface markers, such as FAP^108^ and by inducing cell death through inhibition of the Bcl-2 pathway.^95^ Selected drugs and compounds within each category are summarised in Table 2. It should be noted that many of these therapies are currently administered in combination with standard-of-care chemotherapeutic treatments in an attempt to enhance their effectiveness and/or to reduce CAF-mediated chemoresistance.^103,109^ However, besides compounds in preclinical and clinical stages, there is currently no specific CAF-based adjuvant therapy on the market, which is undoubtedly a result of the inability to specifically target CAFs, due to a lack of CAF-exclusive surface markers and the functional heterogeneity within the CAF population.^11^

**Table 2:** Clinical strategies to modulate CAF activation in the TME for anticancer therapeutic intervention.

Drug/compound	Cancer type	Mechanism	Clinical stage	Reference(s)
Blocking CAF activation		Relevant life cycle stages: CAF priming, CAF maturation and mature CAF		
Y-27632, Fasudil (HA-1077)	PDAC	Inhibits Rho/ROCK pathway	Preclinical	^100^
NT157	Colorectal	Targets STAT3 and IGF-1R pathways	Preclinical	^101^
Targeting the CAF hyperproliferative phenotype		Relevant life cycle stages: CAF maturation and mature CAF		
Tranilast	Lymphoma, lung	Anti-fibrotic agent that suppresses proliferation and TGF-β release	Preclinical	^102^
Pirfenidone	Pancreatic, NSCLC	Anti-fibrotic agent that suppresses proliferation and downregulates TGF-β, PDGF and collagen synthesis	Phase 1 (NCT03177291)	^103^
Targeting mature CAF function		Relevant life cycle stages: CAF maturation and mature CAF		
Metformin	Ovarian	Inhibits IL-6 secretion by suppressing NF-κB signalling	Preclinical	^105^
Imatinib	Cervical	Blocks PDGF receptors	Preclinical	^106^
Sonidegib (LDE225)	Triple-negative breast	Inhibits Hedgehog signalling through SMO inhibitor	Phase 1 (NCT02027376)	^109^
Val-boroPro (Talabostat)	Colorectal	Inhibits FAP enzymatic activity	Phase 2	^119^
Pasireotide (SOM230 analogue)	PDAC	Inhibits mTOR/4E-BP1 protein synthesis pathway	Phase 1 (NCT01385956)	^120^
Losartan	Breast	Decreased collagen I synthesis	Preclinical	^121^
AMD3100	Gastric	Inhibits CXCL12/CXCR4 signalling	Preclinical	^122^
Inactivating the CAF phenotype		Relevant life cycle stages: mature CAF, senescent CAF and CAF inactivation		
AC1MMYR2	Breast, glioblastoma and gastric	Inhibits microRNA-21 maturation via NF-κB/miR-21/VHL axis	Preclinical	^123^
Dasatinib	Lung	Inhibits PDGFR	Preclinical	^124^
Vitamin D receptor ligand (Calcipotriol)	PDAC	Binds to master transcriptional regulator of CAFs	Preclinical (NCT02030860)	^97^
All*-trans*-retinoic acid	PDAC	Biomechanical reprogramming through an ATRA-dependent downregulation of actomyosin contractility via the RARβ/MLC2 pathway	Phase 1 (STAR_PAC: NCT03307148)	^125^
Ruxolitinib	Head and neck, lung and breast	Inhibits JAK/STAT pathway and DNA methyltransferase activity	Phase 2 (NCT03153982)	^60^
Minnelide	PDAC	Suppresses TGF-β signalling pathway	Preclinical	^107^
Depleting the CAF population		Relevant life cycle stages: mature CAF, senescent CAF and CAF inactivation		
aFAP-PE38	Breast	Immunotoxin that binds to FAP	Preclinical	^108^
Navitoclax (ABT-263)	Cholangiocarcinoma	BH3 mimetic that initiates cell death by inhibiting Bcl-2 proteins	Preclinical	^95^

*ATRA* all*-trans*-retinoic acid, *BH3* Bcl-2 homology domain 3, *CAF* cancer-associated fibroblast, *CXCL12* CXC motif ligand 12, *CXCR4* CXC motif chemokine receptor 4, *IGF-1R* insulin-like growth factor type 1 receptor, *IL-6* interleukin-6, *FAP* fibroblast activation protein, *JAK* Janus kinase, *MLC2* myosin light chain 2, *mTOR* mammalian target of rapamycin, *NF-κB* nuclear factor κB, *NSCLC* non-small-cell lung cancer, *PDAC* pancreatic ductal adenocarcinoma, *PDGF* platelet-derived growth factor, *RARβ* retinoid acid receptor β, *ROCK* Rho kinase, *SMO* smoothened, *STAT3* signal transducer and activator of transcription 3, *TGF-β* transforming growth factor-β, *VHL* von Hippel–Lindau

## Outlook

The growth and phenotypic changes that occur in tumour cells during disease progression are concurrent with, and enabled by, a co-evolving tumour stroma. In this review, we have outlined the properties of fibroblasts at different stages in their life cycle within this stroma, from fibroblast quiescence in homoeostasis to CAF senescence, and highlighted the parallels between activated states in acute injury and in chronic wounds/tumours. Based on this life cycle, we have also highlighted the therapeutic opportunities available to leverage CAF inactivation to achieve CAF depletion from tumours. Similar to the situation in chronically fibrotic tissue, in which a critical mass of myofibroblasts is responsible for extreme levels of matrix deposition (which ultimately leads to the loss of organ function), understanding how to interfere with the recruitment and proliferative mechanisms that lead to increased fibroblast densities in the TME has the potential to be hugely beneficial, as proliferation of CAFs leads to an exponential amplification of the amount of pro-tumorigenic interactions present within the TME.

The overview presented here highlights that the life cycle of a CAF is strewn with knowledge gaps, particularly in its temporal trajectory (Fig. 2). Studies examining fibroblast activation in preinvasive tumours suggest that initial activation must occur very early on in tumour progression, although the mechanisms of this fibroblast priming are unclear. Furthermore, provided that the stages of myofibroblast activation in wound healing are recapitulated in tumour stroma, it remains uncertain how the transient entity of protomyofibroblasts fits into this picture, both chronologically during recruitment, and spatially at increasing distances from the stiffened, desmoplastic tumour lesion. In addition, biomarkers for identifying and targeting fibroblasts during their maturation period are currently not available, although they could have interesting therapeutic implications for inactivating fibrotic stroma. Similarly, in invasive disease, although a consensus exists on the causal relationship between the persistence of activating signals in the cancer wound and the inability of CAFs to undergo inactivation, the mechanism responsible for this within CAF cells is unknown—albeit of great interest for therapeutic purposes.

Our review also emphasises that CAF heterogeneity represents another challenging aspect of tumour biology. The concurrent existence of CAFs with different properties within the TME might depend on the origin of the precursor cell or the evolutionary stage or clonal composition of the TME. At the same time, myofibroblasts/CAFs have been described as cellular entities of a particular functional state, rather than as a singular cell type.^110^ These views imply the convergence of multiple progenitor populations towards a specific set of functionalities within the TME, such as enhanced contractility and protein synthesis. They also raise the question of whether current research efforts are merely capturing snapshot data from tumours, which in reality harbour a much wider hierarchical network of CAF maturation stages. Future studies aiming at revealing the ‘differentiation hierarchy’ of CAFs during tumour evolution must focus on stage-specific and stage-matched in vitro models of CAF–cancer cell co-cultures and untangle intratumoural heterogeneity on a single-cell or CAF subpopulation basis, as well as answering the question of whether in vitro culture approaches are supporting the full spectrum of CAFs present in vivo, and if so, which of these types of CAF might be clinically relevant.

The unknowns scattered throughout the CAF life cycle represent challenges for therapeutic intervention. Understanding these aspects could have implications for therapies that aim to reduce the presence of activated, pro-tumorigenic CAFs in tumour stroma. Such therapies could act on CAFs during their maturation phase, halting their differentiation into fully and irreversibly activated fibroblasts, and thereby cutting off further supply of CAFs for the growing tumour. Finally, a paramount consideration for any CAF-centred therapy will be the question of which part of the CAF spectrum remains unaffected by, or is specifically induced by, a given treatment. Given both the pro- and anti-tumorigenic potential of CAFs, understanding how to appropriately modulate these phenotypes at a given tissue/cancer stage will probably be critical in determining the failure or success of a fibroblast-targeting therapy.

## Data Availability

Not applicable.
